# Retrospective analysis of medium-term outcomes following anterior lumbar interbody fusion surgery performed in a tertiary spinal surgical centre

**DOI:** 10.1308/rcsann.2023.0082

**Published:** 2024-03-13

**Authors:** T Srirangarajan, K Eseonu, B Fakouri, P Liantis, P Panteliadis, J Lucas, T Ember, M Harris, M Tyrrell, B Sandford, JR Panchmatia

**Affiliations:** ^1^ Guy’s and St Thomas’ NHS Foundation Trust; ^2^ King’s College Hospital NHS Foundation Trust

**Keywords:** ALIF, Degenerative Spine, Scoliosis, Spondylolisthesis, Spine Complications, Lumbar Fusion

## Abstract

**Introduction:**

Anterior lumbar interbody fusion (ALIF) can treat spondylolisthesis, degenerative disc disease and pseudoarthrosis. This approach facilitates complete discectomy, disc space distraction, indirect decompression of neural foramina and placement of large interbody devices. Several intra- and postoperative complications can be attributed to the anterior approach: vascular/visceral injury, hypogastric plexus injury and urogenital consequences. Spine-specific complications include implant migration, graft failure, pseudoarthrosis and persistent symptomology.

**Methods:**

This retrospective study reviewed patient demographics, medium-term outcomes and complication rates following ALIF surgery performed over a 5-year period. A total of 110 consecutive patients had undergone ALIF surgery at a single tertiary spinal centre. The database was reviewed with a primary outcome of identifying postoperative 90-day complications and whether a revision anterior operation was required after primary ALIF.

**Results:**

No patients required revision anterior operation after their primary ALIF surgery by final follow-up. Out of 110 patients, 11 (10%) recorded a complication attributed to the anterior stage of their operation within 90 days.

**Conclusions:**

Our 90-day complication rate of 10% lies within the 2.6% acute complication and 40% overall complications rates described in previous literature. The risk of vascular/visceral injury is significant (3%) and we recommend that ALIF be performed as a dual surgeon procedure with a vascular-trained access surgeon accompanying the spinal surgeon. ALIF is a valid revision surgical option for failed posterior approaches leading to complications such as pseudoarthrosis. In our sample, 89% of patients were managed with posterior fixation to augment the anterior fusion as, biomechanically, this is a proven construct.

## Introduction

Anterior lumbar interbody fusion (ALIF) was described in 1932 by orthopaedic surgeon Norman Capener.^1,2^ ALIF remains a popular approach to treat conditions including spondylolisthesis, degenerative disc disease, pseudarthrosis, neoplasia and discitis.

The anterior approach allows direct visualisation of the anterior column. This facilitates a complete discectomy and effective disc space distraction, leading to indirect decompression of the neural foramina and placement of a large interbody device.^3,4^ The ALIF can also effectively restore ‘normal’ anatomical parameters by achieving lumbar lordosis and balance in the coronal and sagittal planes.^5–7^ ALIF can be used in isolation, or as part of a combined anterior–posterior construct.

A number of intra and postoperative complications have been reported following ALIF surgery.^8,9^ Whereas rates of infection are relatively low, vascular and visceral injury can occur during mobilisation of the great vessels.^10^ Anterior exposure of the lower lumbar spine can lead to injury of the hypogastric plexus of nerves, with urogenital consequences including retrograde ejaculation and impotence.^11,12^ Spine-specific complications include implant migration, graft failure, pseudoarthrosis and persistent symptomology.^3,13^

This retrospective study aimed to review patient demographics, perioperative variables and medium-term complication and reoperation rates following ALIF surgery performed over a 5-year period at a tertiary spinal surgical unit in London, UK.

## Methods

### Study design

This retrospective study interrogated a prospectively compiled database. We reviewed data concerning 110 consecutive patients who had undergone ALIF surgery of 162 spinal levels at a single tertiary spinal centre between 1 August 2015 and 31 March 2020. Surgery was performed by one of a group of five fellowship-trained spinal surgeons.

The variables reviewed were:

• patient demographic information;
• pre- and postoperative haemoglobin and creatinine;
• perioperative events/complications;
• 90-day clinic follow-up outcomes;
• implant selection/bone graft use.

Analysis of patient demographic and background information was reviewed using the following variables:

• body mass index (BMI);
• past medical history;
• American Society of Anesthesiologists (ASA) physical status;
• anticoagulant use and immunocompromised patient.

The selection criteria were as follows: degenerative lumbar spinal disease and spinal deformities with a minimum follow-up period of 12 months. Neoplastic, traumatic and infectious cases were excluded from this study.

A perioperative complication was defined as any unexpected adverse event that occurred during the procedure. An early postoperative complication was defined as any unexpected adverse event occurring within 6 weeks of the index procedure. Additional interventions needed following the adverse event were recorded. Complications were subdivided into surgical or medical categories and those occurring in or out of hospital.

We reviewed the indications for surgery and whether the ALIF was a primary or revision procedure. Intraoperative (surgical) time and number of operated spinal segments were also noted.

### Surgical technique

The ALIF approach has been described in detail in the existing literature.^14^ All patients underwent an open ALIF using an anterior approach to the lumbar or lumbosacral spine. Surgery was performed via a left-sided extraperitoneal approach with the support of one of the two vascular access surgeons. Retraction of vasculature and soft tissues was performed with use of either a Synthes Synframe or Nuvasive Retractor System. The incision varied on the approach level and the number of levels performed, with a mini-Pfannenstiel incision used for access to the L5–S1 level and a midline vertical incision used for other levels and multilevel approaches.

The disc level(s) were confirmed radiologically and skin incisions and planes of exposure planned immediately before skin preparation and draping. A vertical paramedian sheath incision and medial-to-lateral reflection of the rectus was employed routinely to expose the anterior preperitoneal layers.

**Figure 1:**
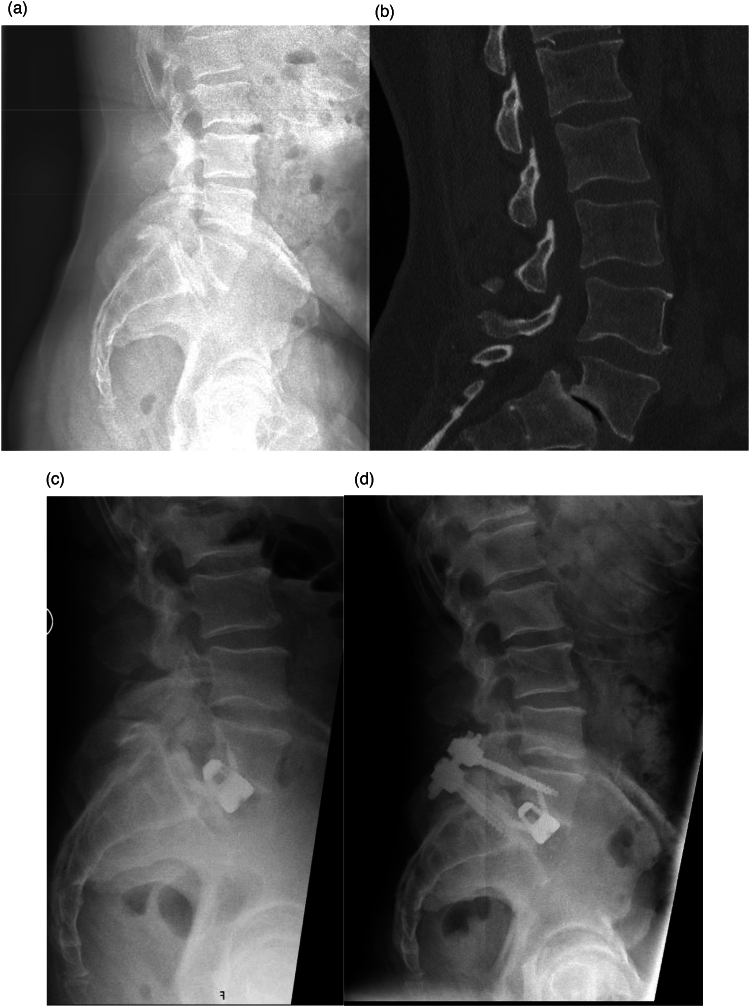
Preoperative and postoperative imaging of a patient with L5/S1 grade 3 spondylolisthesis. (a) Preoperative sagittal lumbar spine radiograph. (b) Preoperative sagittal lumbar spine CT scan. (c) Postoperative first stage ALIF sagittal lumbar spine radiograph. (d) Postoperative second stage posterior lumbar fusion sagittal radiograph. ALIF = anterior lumbar interbody fusion; CT = computed tomography.

An extraperitoneal plane was developed anteriorly, laterally and then posteriorly, with positive identification and preservation of the left ureter and iliac vessels. The peritoneal sac and attached ureter were retracted medially. Disc spaces were exposed by mobilisation of the left common iliac vein with division of branches as necessary. Mobilisation was to the left with division of median sacral vessels for L5/S1 and to the right with division of the ascending lumbar vein(s) for L4/5. Once exposed, disc level was confirmed radiologically before disc removal and end plate preparation. A standalone Nuvasive Brigade polyether ether ketone (PEEK) or BASE (Titanium) ALIF cage was packed with bone graft, inserted and fixed with integral screws into the adjacent cranial and caudal endplates. Before closure further intraoperative radiographs were obtained to ensure correct cage placement in the sagittal and coronal planes (Figure 1).

The ALIF surgery was followed up with either a single or second-stage posterior fixation using either a percutaneous minimally invasive technique or an open technique depending on the number of levels that the fixation spanned.

### Postoperative care

Patients followed a standardised ward-based rehabilitation programme. All patients were encouraged to walk within 24h of surgery. Postoperative venous thromboembolism (VTE) prophylaxis was managed on a case-by-case basis due to the concerns of postoperative bleeding, which can cause complications such as epidural haematoma. Patients were managed with mechanical VTE prophylaxis using intermittent pneumatic calf compression devices until they were able to walk.

## Results

### Patients

A total of 110 patients underwent ALIF surgery between 1 August 2015 and 31 March 2020. Mean follow-up was 25 months (range 13–50.4 months), 42 patients were male (38%) and 68 were female (62%); 11% of patients were smokers. Average and median BMI was 28 (range 17–40) (Table 1) and average age was 47.8 years (range 18–77). A total of 162 spinal levels were operated on; 63 underwent single-level operations and 47 underwent multiple-level surgery (Table 2). The L4–L5 and L5–S1 disc spaces were the most commonly operated levels, comprising 37% (60/162) and 50% (82/162) of operated levels, respectively (Table 3).

**Table 1:** Patient characteristics and diagnosis

Summary of patient characteristics	
Number of patients	110
Total levels of fusion	162
Mean age in years (range)	47.8 (18–77)
Gender (male: female)	42: 68
Mean follow-up (months)	25 (13–50.4)
**Diagnosis**	
Degenerate disc disease	44
Spondylolisthesis	43
Scoliosis	12
Mixed pathology	15

**Table 2:** Operated number of intervertebral levels and anatomical level of operation

Number of intervertebral levels	*n*	%
1	63	57
2	37	34
3	9	8
4	1	0.9
Total number of cases	110	
Operative level	*n*	%
L3-4	20	13
L4-5	60	37
L5-S1	82	50
Total number of levels	162	

**Table 3:** Operation performed: primary vs revision, single vs two stage

Operation performed	
Primary procedure	
ALIF	3
Single stage (anterior and posterior)	17
Two stage (anterior then posterior)	56
Total	77
Revision procedure	
ALIF	9
Single stage (anterior and posterior)	5
Two stage (anterior then posterior)	20
Total	33

ALIF = anterior lumbar interbody fusion.

### Primary outcomes

No patients required a revision anterior procedure after their primary ALIF surgery by final follow-up. Of 110 patients, 11 (10%) had a complication attributed to the anterior stage of their operation within 90 days. The average BMI of these patients was 27.5.

Three (2.7%) patients reported new leg pain following the ALIF procedure. Upon further assessment, these patients demonstrated clinical and radiographical findings of postsurgical neuritis and were given nerve root blocks that helped improve their pain. None of these patients required further surgical intervention.

### Surgical complications

Two (1.8%) patients were reported to have postoperative intra-abdominal retroperitoneal complications. Both patients had undergone L4/5 and L5/S1 two-level ALIF. Neither patient was on preoperative anticoagulants. One patient developed a retroperitoneal lymphatic collection, which was drained under computed tomography guidance. The patient went on to recover without any further surgical intervention. The second patient was found to have a retroperitoneal collection secondary to lymphatic injury that was managed expectantly and went on to resolve.

Two (1.8%) patients sustained intraoperative complications. One patient sustained an intimal tear of the iliac artery during the approach that required vascular repair, without further consequence by final follow-up. The second patient had an intraoperative rise in their lactate to 7mmol/l. This prompted the suspicion of visceral injury, and an exploratory laparotomy was performed, which did not yield any obvious intra-abdominal cause. The patient was resuscitated and lactate recovered to normal levels while they were monitored in the intensive therapy unit (ITU) for one day. The patient did not sustain any further complications. A total of 20 (18%) patients were given cell salvage blood transfusion intraoperatively; 12 of these patients were undergoing single-stage anterior and posterior operations.

### Medical complications

Three (2.7%) patients were found to have symptomatic postoperative anaemia following their ALIF surgery and required 2 units of packed red cell transfusion. None of these patients were on preoperative anticoagulants. One (0.9%) patient developed postoperative pneumonia and required a course of oral antibiotics to treat their infection.

### Perioperative variables

Mean surgical time for a primary ALIF performed either as an isolated primary ALIF or the anterior stage of a two-stage (anterior then posterior) procedure was 223min (range 139min–356min). These patients included 122 operated segments with an average of 154min per segment (range 62min–360min) (Table 4).

**Table 4:** Average operative time

Procedure	Average operative time (range)	Average operative time per segment (range)
PRIMARY		
ALIF	223min (130–356min)	154min (62–360min)
Single stage (anterior and posterior)	315min (175–704min)	237min (97–365min)
Revision		
ALIF	220min (143–332min)	177min (75–302min)
Single stage	322min (170–487min)	201min (150–243min)

ALIF = anterior lumbar interbody fusion.

Mean surgical time for a primary single level ALIF as an isolated primary ALIF or anterior stage of a two-stage procedure was 184min (range 130min–270min) (Table 5).

**Table 5:** Average operative time per spinal level segment

Procedure	Average operative time (range)
Primary ALIF	
Single level	184min (130–270min)
Two level	234min (160min–323min)
Multilevel	342min (187–540min)
Revision ALIF	
Single level	223min (143–302min)
Two level	217min (151–332min)
Multilevel	No multilevel revision procedures

ALIF = anterior lumbar interbody fusion.

Mean pre- to postoperative haemoglobin blood tests demonstrated a drop of haemoglobin: −17g/dl (+31 to −60g/dl). Twenty patients underwent intraoperative cell salvage transfusion. Four patients required red blood cell transfusion in the 24h postoperatively. The average operative time of the patients requiring postoperative transfusion was 287min. Of the patients receiving a blood transfusion, the average drop in haemoglobin after their ALIF surgery was −34g/dl (Table 6).

**Table 6:** Average postoperative haemoglobin for ALIF

ALIF procedures (excluding single stage)	Average haemoglobin (range)
Single level	−15g/dl (+31 to −60g/dl)
Two level	−20g/dl (+16 to −44g/dl)
Multilevel	−18g/dl (+9 to −46g/dl)

ALIF = anterior lumbar interbody fusion.

Mean pre- to postoperative estimated glomerular filtration rate was +0.9ml/min/1.73m^2^ (range −60 to +62ml/min/1.73m^2^).

We measured total inpatients days (excluding patients who had to wait as an inpatient for second-stage posterior surgery, which would have prolonged their inpatient stay from factors not attributable to the ALIF surgery). Mean inpatient stay in hospital was 3.7 days (range 2–8).

Postoperatively, 11 (10%) patients required intensive care monitoring. These patients all stayed on ITU for one day duration only for extra monitoring, and none required vasopressor or ventilatory support (Table 7).

**Table 7:** Average length of stay per spinal level of operation

ALIF procedures (excluding single stage anterior-posterior)	Average length of stay (range)
Single level	3.3 days (2–8 days)
Two level	4 days (3–7 days)
Multilevel	6 days (5–7 days)

ALIF = anterior lumbar interbody fusion.

Surgical time, blood loss and length of inpatient stay were all increased for patients undergoing two-level and multilevel surgery.

### Implant selection/bone graft usage

Our unit transitioned from using PEEK to Titanium BASE plates between 2017 and 2018. A total of 34 (31%) patients were instrumented with a PEEK cage, while 74 (67%) patients had BASE titanium cage. One patient had iliac crest bone graft as their interbody space was too small to fit a cage. Another patient was fitted with the Depuy Synthes Synfix cage. A total of 97 (88%) patients had bone graft (Nuvasive ATTRAX +/− DBX Putty) packed into the cage. Of these patients, 11 had InductOS Bone Morphogenetic Protein (BMP) 2 to aid fusion.

## Discussion

The ALIF is a well-recognised lumbar spinal surgical technique in both primary and revision settings.^3^ The anterior approach to the lumbar spine has the advantage of optimal disc space clearance, allowing the insertion of a large implant that can effectively restore sagittal anatomy.^15^ Aggressive correction of the lordosis and restoration of disc height contributes to indirect neural decompression by achieving increased foraminal dimensions.^16^ It also places the cage graft in compression and the increased implant to vertebral body contact area and anterior vascularity has demonstrated high fusion rates when comparing with posterior and postero-lateral approaches.^17,18^

### Complications

The 90-day complication rate of 10% in our cohort lies within the 2.6% acute complication and 40% overall complications rates described in published literature.^8,10,19–21^

Confirmed intraoperative abdominal complications occurred in three (3%) patients in our study. This included one patient with a vascular intimal tear and two patients with intra-abdominal collections believed to be retroperitoneal lymph formation. Sasso *et al* describe intraoperative vascular injury occurring at a rate ranging from 0.4% to 3.1% and it was found that threaded implants required larger instruments and retractors that could cause vascular injury when compared with instruments used in nonthreaded implants.^19^ Rajaraman *et al* described a 6% vascular injury and an overall 38% general surgical-related complication rate following ALIF.^10^ Momin *et al* describe similar intraoperative vascular injury rate of 5% as well as a 0.6% rate of retroperitoneal haematoma.^21^ Rao *et al* identified retroperitoneal haematoma as a serious and potentially fatal complication. They highlighted three patients (2.4%) who developed retroperitoneal haematoma in a retrospective series of ALIF patients, of whom two required exploratory surgery.^22^

Surgery-related visceral injury is rare in spinal surgery but is a particular concern with the anterior approach. The literature describes a 0.9%–1.6% rate of injury to the bowel/intraperitoneal structures during the ALIF procedure (related to the approach in the majority of cases) and in all the reported cases an urgent surgical exploratory laparotomy was conducted.^10,21^

Wound complications can range from minor superficial wound infections requiring short-term antibiotics to major infective collections causing systemic upset and even pressure-related neural compromise.^20^ We did not note any complications of the anterior wound in our cohort. Reported complications from anterior abdominal wounds following ALIF include infection (2%) and wound dehiscence (1%).^21^ Published work has reported similar anterior wound complication incidences ranging from 1% to 4%.^23^

### Approach

The approach to the anterior lumbar spine is divided broadly into a transperitoneal and a retroperitoneal approach. The transperitoneal approach allows direct access to the lower lumbar levels of L4/S1 but risks visceral injury and complications such as paralytic ileus and retrograde ejaculation from injury to the hypogastric plexus.^24,25^ The retroperitoneal approach is favoured from the left side to help protect and avoid retraction of the inferior vena cava, allowing mobilisation of the aorta and access to high lumbar levels extending from L2/S1.^24,26^ Ureteric injury has been described in retroperitoneal approaches, as the posterior peritoneum is swept forward during the approach.^27^

The approach in our cohort was performed by a vascular access surgeon via a retroperitoneal approach. No bowel or genitourinary complications were noted. We are in agreement with previous work by Mobbs *et al*, which concluded that major harm can be minimised by the implementation of a team-based approach, with a vascular-trained access surgeon accompanying the spinal surgeon.^28^

### Revision surgery

ALIF is a valid revision surgical option for failed posterior approaches leading to complications such as pseudoarthrosis, necessitating fusion below the level of L4.^29^ All 33 revision procedures we investigated were due to failed posterior surgery, with patients complaining of persistent back pain and radiographical appearances of progressive degenerative lumbar spine disease. ALIF provides an approach through virgin tissue, avoiding the dissection and retraction of scarred perineural tissue and dura. ALIF also avoids complications associated with revision posterior lumbar surgery, including dural tear and neurologic injury.^30,31^

### Posterior fixation

In our sample, 89% of patients were managed with posterior fixation to augment the anterior fusion during their surgeries. From the remaining patients, nine had already had posterior fixation from a previous operation and three patients had a diagnosis of degenerative disc disease and underwent standalone single-level ALIF primary surgery. Biomechanical studies have suggested that standalone interbody cages are stable in flexion and lateral bending, but that stability in extension and rotation is poor. Where standalone fixed cages are insufficient, it is necessary to include more rigid posterior fixation with or without adjuvant anterior plating.^32^

### Operative time and hospital length of stay

Our average operative time for an ALIF was 223min. This is comparable with the mean operative time described by Upadhyayula *et al* of 198min for an ALIF and 210min for a transforaminal lumbar interbody fusion. This study also described a similar hospital length of stay to that of our cohort average of 3.7 days.^33^

### Cage type and bone graft

The Guy’s and St Thomas’ NHS Trust underwent a transition from PEEK to titanium baseplate cages. Titanium is a material that has been demonstrated to have better osteointegration than standalone PEEK cages.^34^ PEEK is a material with similar modulus of bone and has demonstrated reduced rates of subsidence.^35,36^ The use of demineralised allograft and BMP has been shown to improve fusion rates. BMP is not without its controversial complications of ectopic bone formation and osteolysis.^37,38^ We did not experience these complications and found all patients went on to achieve fusion throughout their follow-up.

## Conclusion

Our retrospective study supports the role of ALIF in both primary and revision settings in the lumbar and lumbosacral spine. Most commonly operated levels were L4/5 and L5/S1, which corresponds with the most commonly identified vascular anatomy in the lower lumbar spine.

We report a rate of acute and medium-term complications and reoperation comparable with rates in the existing literature. Only four (3.6%) cases in our series experienced vascular injury or retroperitoneal collection and none of these led to negative long-term sequelae. All cases in our cohort were approached via a retroperitoneal method and our study supports this as a safe approach that minimises the rate of visceral and vascular injury.

This study adds to the body of evidence supporting the use of ALIF as a fusion strategy in the armamentarium of the spinal surgeon in the management of degenerative disc disease, spondylolisthesis and scoliosis in the primary and revision settings. Further work is needed to identify the long-term complication and reoperation profile in this cohort.
